# Clinical benefits and current challenges of photon-counting detector CT in vascular imaging

**DOI:** 10.1093/radadv/umag021

**Published:** 2026-04-15

**Authors:** Ahmed O El Sadaney, Felix E Diehn, Prabhakar Shantha Rajiah, Ian Mark, Norbert G Campeau, Adam T Froemming, Francis I Baffour, Kelly K Horst, Lifeng Yu, Shuai Leng, Cynthia H McCollough, Joel G Fletcher

**Affiliations:** Department of Radiology, Mayo Clinic, Rochester, MN, 55902, United States; Department of Radiology, Mayo Clinic, Rochester, MN, 55902, United States; Department of Radiology, Mayo Clinic, Rochester, MN, 55902, United States; Department of Radiology, Mayo Clinic, Rochester, MN, 55902, United States; Department of Radiology, Mayo Clinic, Rochester, MN, 55902, United States; Department of Radiology, Mayo Clinic, Rochester, MN, 55902, United States; Department of Radiology, Mayo Clinic, Rochester, MN, 55902, United States; Department of Radiology, Mayo Clinic, Rochester, MN, 55902, United States; Department of Radiology, Mayo Clinic, Rochester, MN, 55902, United States; Department of Radiology, Mayo Clinic, Rochester, MN, 55902, United States; Department of Radiology, Mayo Clinic, Rochester, MN, 55902, United States; Department of Radiology, Mayo Clinic, Rochester, MN, 55902, United States

**Keywords:** photon-counting-detector CT, angiography, coronary artery disease, atherosclerosis, run-off CTA, peripheral vascular diseases, cerebrovascular disorders, cerebrospinal fluid-venous fistula

## Abstract

Photon-counting detector CT (PCD-CT) directly detects individual photons and measures their energies, enabling the acquisition of multi-energy data from a single x-ray source with higher spatial resolution, thereby allowing visualization of smaller arteries and veins and other important imaging features not seen at conventional CT. It also substantially reduces calcium and stent blooming artifacts. Appropriate patient selection and task-based optimization of acquisition, reconstruction, and interpretation are critical to realize these benefits. Vascular imaging examinations for which early evidence shows that PCD-CT demonstrates higher diagnostic performance compared to conventional CT include coronary CT angiography (CTA) for evaluation of partially calcified plaques or stents, peripheral runoff CTA, detection of cerebrospinal fluid-venous fistulas, and head CTA for evaluation of small intracranial vessels and aneurysms. Current limitations include excessive image noise with the sharpest reconstruction kernels and challenges when adapting conventional CT protocols (eg, requiring sharper kernels, thinner slices, higher matrices, tailored scan modes) to achieve time-efficient image reconstruction and hanging protocols. New solutions and directions will include deep-learning-based image noise reduction, improved spectral separation, strategic triage of specific patient populations to PCD-CT systems, and subspecialty clinical practice recommendations for this new technology.

**Abbreviations** 

AI = artificial intelligence; CSF = cerebrospinal fluid; CVF = cerebrospinal fluid-venous fistula; DLR = deep-learning-based denoising and reconstruction; EID = energy-integrating detector; ICA = invasive coronary angiography; IR = iterative reconstruction; PCD = photon-counting detector; UHR = ultra-high resolution; VMI = virtual monoenergetic image

**Summary** 

Photon-counting detector CT (PCD-CT) permits visualization of small vessels and important vascular features not seen with conventional CT and improves stenosis assessment in the presence of calcific plaque or stents.

**Essentials** 

- PCD-CT can visualize small arteries and veins (eg, cerebrospinal fluid-venous fistulas) and vascular morphological features not seen at conventional CT.
- PCD-CT angiography reduces calcium and stent blooming artifact, resulting in more accurate luminal stenosis assessment.
- Routine generation of virtual monoenergetic images permits flexibility to adjust iodine dose and reduce artifacts.
- Integration of PCD-CT into clinical practice requires dedicated protocols, technologist training, and intentional patient triage to identify those likely to benefit.

## Introduction

Photon-counting detector CT (PCD-CT) differs from conventional CT systems by using a semiconductor detector that directly converts the energy of incident x-ray photons into electrical signals. Photons are detected individually and sorted into predefined energy bins according to their energy.^1–4^ This approach substantially increases spatial resolution and radiation dose efficiency by eliminating the need for reflective septa, which are used in conventional x-ray detectors to reduce cross-talk from scintillation events and limit detector pixel size. In the current commercially available PCD-CT system, the effective slice thickness is 0.2 mm in high-resolution mode and 0.4 mm in standard mode compared to 0.5–0.6 mm for most conventional CT systems.^3^ Directly recording photon energies allows increased signal for low-energy photons, which are otherwise downweighed by scintillation detectors in conventional CT scanners and permits multi-energy imaging using a single x-ray tube.

Current PCD-CT systems primarily use virtual monoenergetic images (VMIs) as the primary method for image reconstruction to display anatomic and spectral information. VMIs simulate images acquired with a monoenergetic x-ray source: low-energy VMIs (eg, 40–50 keV) accentuate iodine signal (ie, CT attenuation from iodine) for vascular and oncologic imaging, whereas high-energy VMIs (eg, 100–140 keV) reduce calcium blooming and other artifacts. Other multienergy images are also available, such as virtual noncontrast images, which simulate the appearance of a noncontrast image from a contrast-enhanced scan.^5^ VMIs are used in imaging protocols by scanning in a multienergy mode to address multiple challenges associated with a particular diagnostic task.

Since Food and Drug Administration clearance in September 2021, clinical studies have demonstrated that PCD-CT provides distinct advantages for vascular imaging. This review aims to guide radiologists in using PCD-CT for specific diagnostic tasks, highlighting evidence to identify patients most likely to benefit from this technology and outlining strategies for integrating it into large, multivendor practices.

## Current and emerging diagnostic tasks in PCD-CT vascular imaging

PCD-CT angiography (PCD-CTA) benefits many diagnostic tasks that are performed poorly or suboptimally with conventional CT, particularly in patient groups prone to its limitations (eg, dense calcifications, stents). Radiologists need to understand how different PCD-CT images are created and used in clinical protocols to adapt vascular imaging for this new technology. Using a PCD-CT system with protocols designed for conventional CT undermines the ability of PCD-CT to provide new and better information to patient care, so acquisition protocols must be optimized to leverage the increased spatial resolution and improved image quality of multienergy images.

For vascular imaging using PCD-CT, radiologists should aim to maximize spatial resolution while retaining multienergy capabilities from VMIs to increase iodine signal or suppress calcium blooming artifact. Spatial resolution can be increased at PCD-CT by using sharper reconstruction kernels unavailable in conventional CT, higher image matrix sizes (eg, 1024 × 1024 vs. 512 × 512), and thinner slices. Although thin-slice PCD-CT images have less noise than conventional CT owing to the elimination of electronic noise and higher detector sampling, increased image noise from sharper kernels and thinner slices may limit their utility for fine-detail tasks. Therefore, optimizing PCD-CT involves balancing spatial resolution, iodine contrast, and noise for each diagnostic task. Table 1 provides an overview of image acquisition and reconstruction parameters that can be used to maximize spatial resolution and iodine contrast while reducing image noise and artifacts, thereby permitting routine visualization of small vessels that are poorly seen with conventional CT. By manipulating these parameters (eg, to improve spatial resolution or reduce calcium blooming), many of the frustrating limitations of conventional CTA can be overcome. For example, small vessel visualization (eg, small intracranial arteries or cerebrospinal fluid [CSF]-venous fistulas [or CVF]) can be improved by increasing spatial resolution within acceptable noise levels and/or by enhancing iodine contrast within vessels to increase visibility with low-energy VMIs.^6^^,^^7^

**Table 1:** Acquisition and reconstruction parameters used in the current commercial PCD-CT system (Siemens Alpha scanners) and their impact on image quality.

PCD-CT image acquisition and reconstruction parameter		Guidance for image acquisition
**Spatial resolution increase**	Scan mode	UHR scans use a narrow collimation (120 × 0.2 mm) to produce 0.2-mm-thick images. A multienergy UHR mode (96 × 0.2 mm) facilitates VMI and other multienergy images, but at 0.4-mm thickness
	Reconstruction field of view	Smaller is generally better, with the optimal field of view depending on the necessary sharpness and anatomic coverage
	Reconstruction kernel	With the existing manufacturer (Siemens Healthcare), higher numbers are associated with sharper reconstruction kernels and higher spatial resolution; letters indicate other properties designed for spectral applications (eg, “Bv”- kernel has edge-sharpening properties for vascular applications)
	Slice thickness	Noise increases as slice thickness decreases, but to a smaller degree than with conventional CT
	Matrix	PCD-CT supports a 1024 × 1024 matrix, compared to 512 × 512 matrix for most conventional CT systems
	VMI energy level	Sharper when VMI energy level is closer to the mean effective energy (eg, 67 keV VMI at 120 kV). For example, a 40 keV image will be less sharp
**Iodine signal increase**	VMI energy level	Lower energy levels (40–50 keV) increase iodine signal and are often used to increase the conspicuity of small vessels, or to compensate for suboptimal opacification or lower doses of contrast
	Tube potential	Although conventional CT uses lower x-ray tube potentials to reduce radiation dose in smaller patients and increase iodine signal, PCD-CT achieves a similar iodine signal at higher tube potentials; lower x-ray tube potentials at PCD-CT have fewer multienergy options owing to less spectral separation
**Noise decrease**	QIR	Manufacturer’s iterative reconstruction algorithm.^7^ Higher strength settings provide stronger dose reductions but can decrease low-contrast spatial resolution
	Reconstruction kernel	Smoother reconstruction kernels have less noise; some kernels offer inherent noise reduction
	Slice thickness	Thicker slices reduce noise but also z-axis spatial resolution
**Artifact decrease**	Tube potential	Higher values used to improve image quality by increasing spectral separation
	VMI energy level	Higher energy VMIs (higher keV values) reduce calcium blooming and stent-related artifacts
	Reconstruction kernel	Sharper kernels reduce calcium blooming and stent related artifacts
	Iterative metal artifact reduction	Used to reduce metal artifacts (eg, hip prostheses)

Abbreviations: CNN, convolutional neural network; PCD, photon-counting detector; QIR, quantitative iterative reconstruction; UHR, ultra-high-resolution; VMI, virtual monoenergetic image.

Despite the short time that a Food and Drug Administration-cleared clinical PCD-CT system has been available, multiple diagnostic tasks and patient populations in vascular imaging have been shown to benefit from optimal use of the technology. Table 2 catalogues multiple PCD-CTA studies with reference standards that demonstrate the patient benefit of this technology.^8–16^

**Table 2:** Current evidence of diagnostic performance of PCD-CT for various tasks in vascular imaging.

Diagnostic task	Studies (first author, year)	Population	Method of accrual/No. Centers	Refence standard	Comparison modality	Key results
**Coronary artery stenosis**	Shin D. et al. 2025^8^	171 patients (283 stented lesions)	Retrospective, single center	ICA (quantitative)	ICA	Lesion-level sensitivity/specificity of 80.0% and 90.4% for detecting ≥ 50% in-stent stenosis Overall per-lesion accuracy 88.9% and per-patient accuracy of 85.7% No change in AUC between stents <3 mm and ≥3 mm (AUC 0.90 vs. 0.92, *P *= 1.0)
	Vecsey-Nagy et al., 2024^9^	49 patients with visible coronary calcium at EID-CTA (278 stenotic areas [calcified, 202; partially calcified, 51; noncalcified, 25])	Prospective, single center	ICA (quantitative)	Conventional CT	Only 12 patients had ICA. Not a diagnostic accuracy study. Narrower limits of agreement with ICA using PCD-CTA in 12 patients. Significant decreases in estimated percent stenosis scores in the presence of calcified and partially calcified plaque (*P *< .001), with reclassification of CAD-RADs category downward in 24 patients (49%)
	Sakai et al., 2025^10^	903 patients referred for ICA (from 7833 who underwent prior PCD-CTA: 3876; or EID-CTA: 3957)	Retrospective, Single Center	ICA (Quantitative) and subsequent revascularization	Conventional CT	Observational study of patients who underwent PCD-CTA or EID-CTA. Patients were referred for ICA based on CAD-RADS score of 4 or positive CT-derived fractional flow reserve. Quantitative coronary angiography was performed for ICA reference. PCD-CTA decreases referral to ICA compared to EID-CTA (9.9% vs 13.1%; *P *< .001) Specificity in stenosis level analysis was better at PCD-CTA compared to EID-CTA (98.0% vs 93.0%; *P *< .001) Among those who underwent ICA, revascularization was more frequently performed after PCD-CTA (43.4% vs. 35.5%, *P *= .02)
	Hagar et al., 2023^11^	68 patients with severe aortic valve stenosis (965 segments)	Prospective, Single Center	ICA	NA	Prospective study in a challenging cohort of patients (35% of subjects had an Agatston score ≥ 1000; 22% had stents) AUC for per-patient detection of CAD was 0.93 (95% CI: 0.86, 0.99) 0.94 per vessel (95% CI, 0.91-0.98), 0.92 per segment (95% CI, 0.87-0.97). If Agatston score of ≥ 1000, sensitivity/specificity = 93%/70% If stent present, sensitivity/specificity = 100%/83%
	Hagar et al., 2024^12^	18 with 44 coronary stents	Prospective, single center	ICA	NA	Both readers recognized all in-stent stenoses ≥ 50% Sensitivity/specificity for two readers (per stent) 100%/92% and 100% 87%, respectively
**Detection of unruptured intracranial aneurysms (UIAs)**	He et al, 2025^13^	95 participants: 42 cases with confirmed 50 UIAs, and 53 negative cases	Prospective, single center	DSA	Standard and UHR modes	On a per-aneurysm basis, UHR performed significantly better than standard resolution (*P* < .05): Sensitivity: 98.0% vs 72.0%, Specificity: 96.7% vs 86.7% Diagnostic accuracy: 97.3% vs 80.0%
**CSF venous fistula diagnosis**	Schwartz et al., 2024^14^	38 with 11 with definitive, 13 with possible, and 14 without fistula	Retrospective, single center	Unblinded review of both PCD- and EID-CTM with attenuation >70 HU in paraspinal veins in positive scans confirming definite CVF	Conventional CT	The performance for readers 1, 2, and 3 in EID-CT respectively was: Sensitivity of 45%, 36%, and 57% Specificity of 96%, 100%, and 100% The performance for readers 1, 2, and 3 in PCD-CT, respectively, was: Sensitivity of 64%, 55%, and 55% Specificity of 85%, 96% and 93%
**Peripheral artery disease detection**	Ghibes et al., 2024^15^	109 patients	Retrospective, single center	DSA	PCD-CT calcium-removal image reconstruction algorithm (PureLumen; 91 patients)	Overall sensitivity, specificity and accuracy for PCD-CTA in detecting stenosis >60% was 91%; 95% and 93%, respectively, without a calcium-removal image reconstruction algorithm (PureLumen); and 85%, 89%, and 88%, respectively, with a calcium-removal image reconstruction algorithm (PureLumen).
	Augustin et al., 2024^16^	39 patients	Retrospective, single center	DSA with only > 60% stenosis considered positive	Three kernels Bv36, 48, and 56	Sensitivity in comparison between Bv36, 48, and 56 in detecting stenosis over 60% (81.6% vs. 81.5% vs. 81.0%, *P *= .797) Specificity in comparison between Bv36, 48, and 56 in detecting stenosis over 60% (71.1% vs. 76.9% vs. 79.6%, *P *= .067)

Abbreviations: CTA, CT angiography; DSA, digital subtraction angiography; EID, energy-integrating detector; ICA, invasive coronary angiography; PCD, photon-counting detector; UHR, ultra-high resolution; UIA, unruptured intracranial aneurysms.

### Coronary CTA

**Figure 1:**
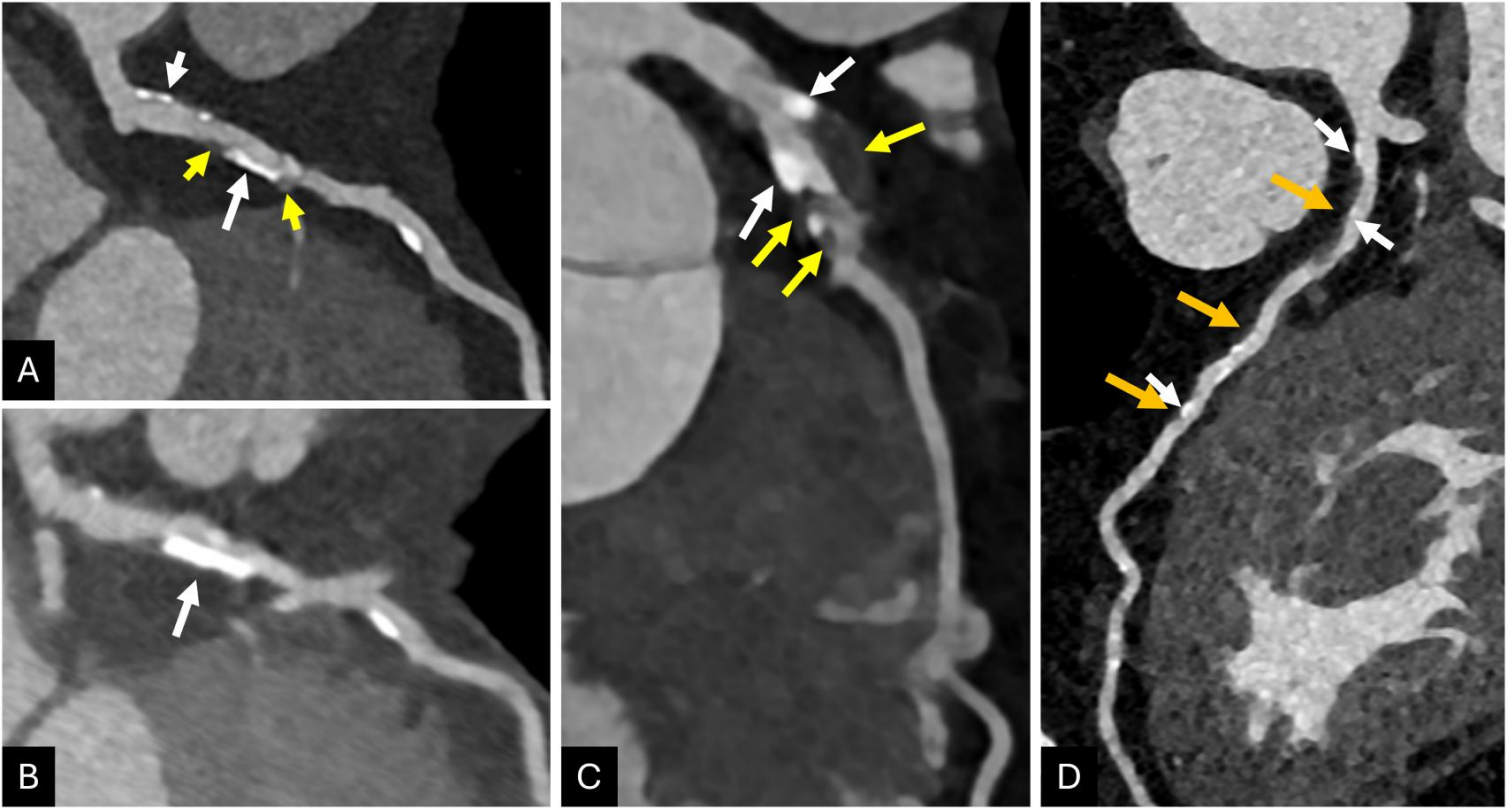
An 82-year-old male underwent 2 coronary CT angiography examinations obtained 2 months apart (A, PCD-CTA; B, EID-CTA), demonstrating a left anterior descending (LAD) artery with multifocal calcified plaque in the proximal and mid segments. Note that on EID-CT (B), focally moderate stenosis is shown adjacent to a calcific plaque (white arrow); however, PCD-CT (A) in the same region shows only mild stenosis (white arrow) and improved visualization of noncalcified (yellow arrow) components. A 77-year-old female underwent PCD-CT coronary angiography (C), showing a large complex plaque including calcified (white arrows) and noncalcified (yellow arrows) components in the proximal LAD with moderate luminal stenosis. A 68-year-old male underwent PCD-CT coronary angiography (D), showing a predominantly soft plaque in the proximal and mid LAD (orange arrows) with spotty calcifications (white arrows). Abbreviations: CTA, CT angiography; EID, energy-integrating detector; PCD, photon-counting detector.

**Figure 2:**
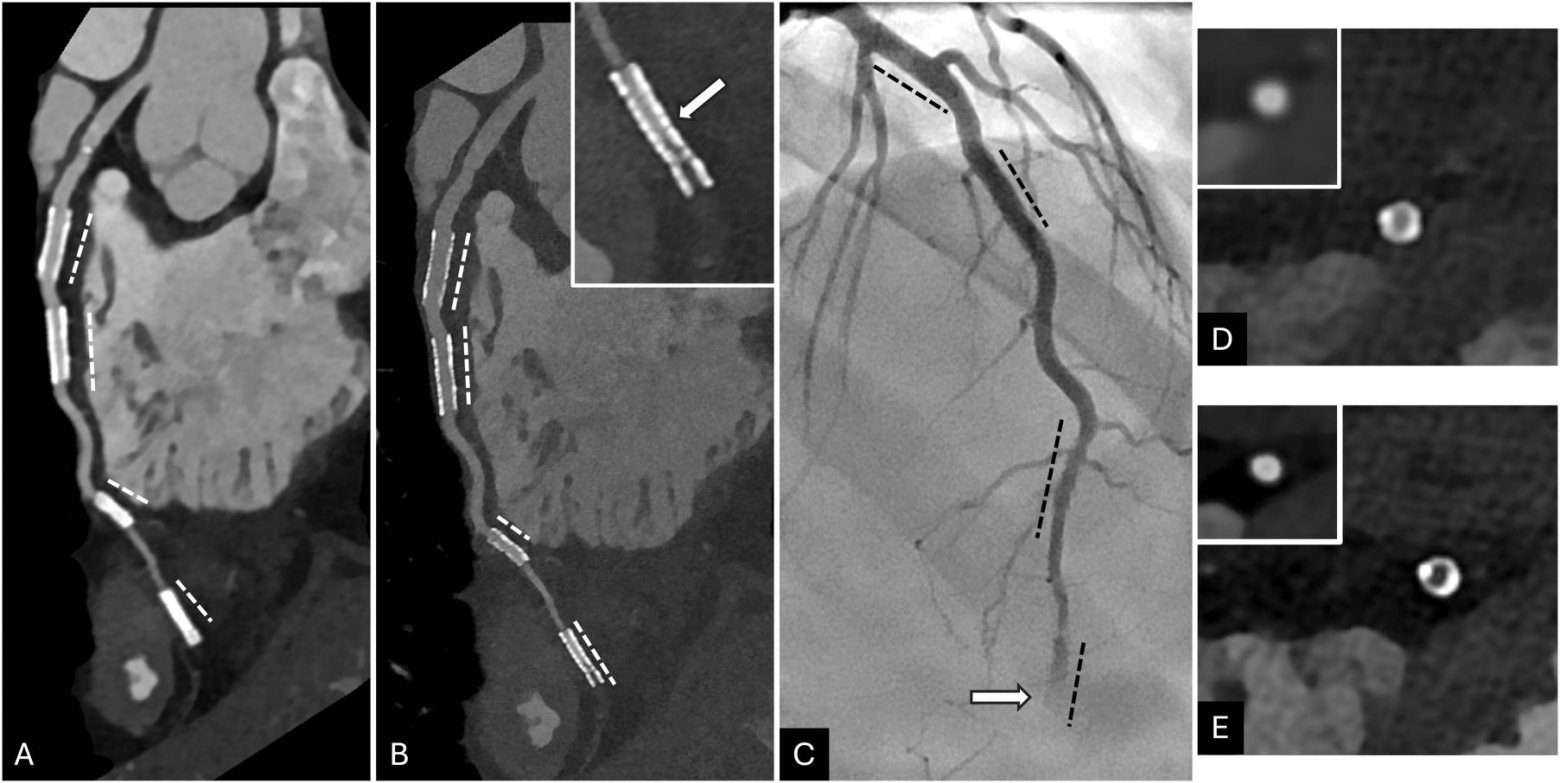
A 36-year-old female underwent PCD-CT coronary angiography examination that was reconstructed with high-resolution parameters with Bv56 kernel. 2-mm slice thickness and 1024 × 1024 matrix (B, D, and E); and parameters mimicking the maximum resolution available on EID-CT with Bv40 kernel, 6-mm slice thickness, and 512 × 512 matrix (A; and insets in D and E). The images demonstrating a left anterior descending (LAD) artery with 4 stents (dashed lines in A, B, and C) with occlusion involving the lower third of the fourth stent (white arrows in B inset and C). The EID-CTA like reconstructions fail to show the lumen in the third and fourth stents (less than 3 mm, A and inset D) and additionally the occlusion (A, and inset E ) that was apparent in high-resolution reconstruction (E) compared to the patent lumen at the proximal part of the stent (D). Abbreviations: CTA, CT angiography; EID, energy-integrating detector; PCD, photon-counting detector.

**Figure 3:**
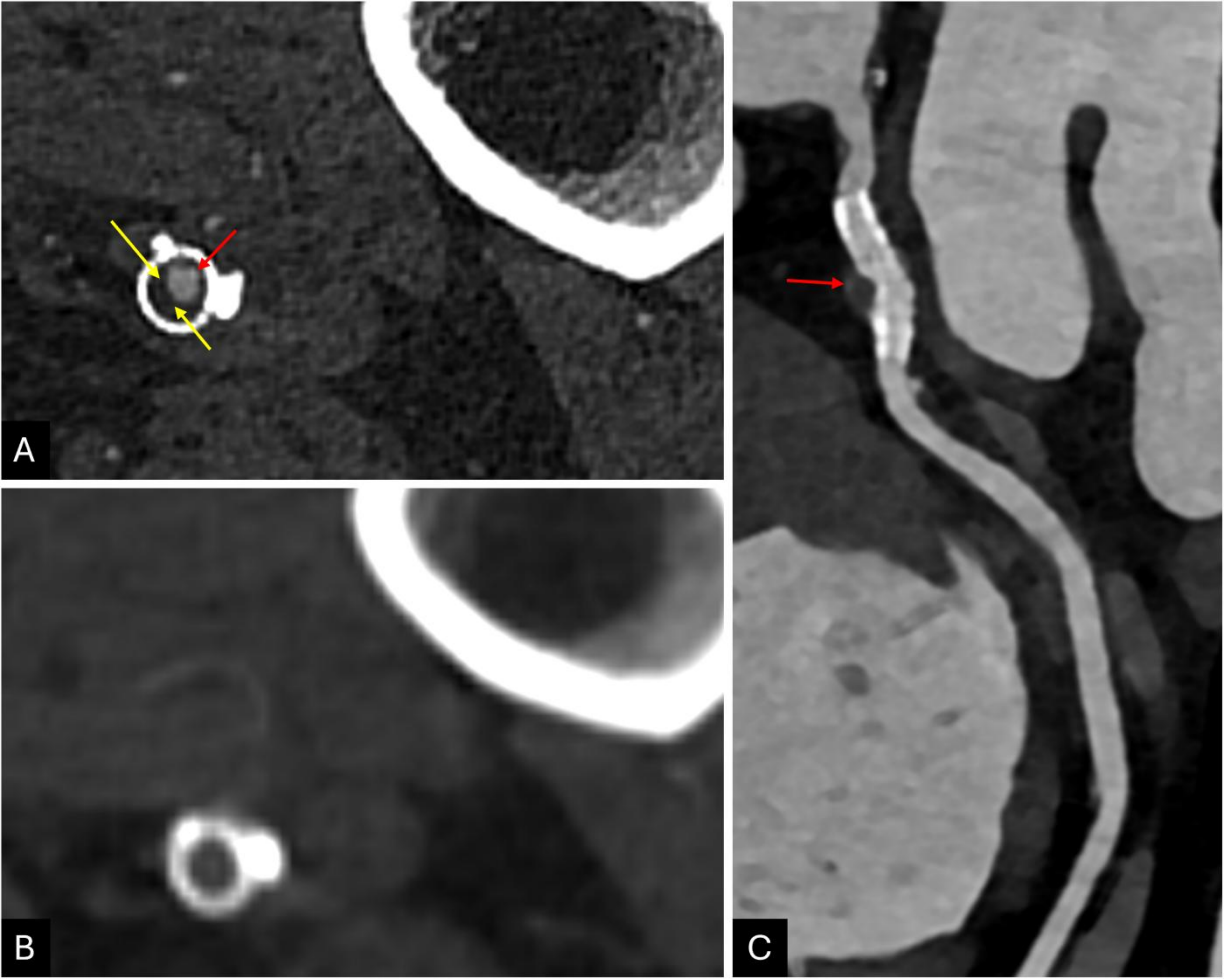
A 64-year-old male with PCD-CTA (A) and EID-CTA (B) runoff study showing a plaque (yellow arrow in A) in a femoral artery stent, stenosing the lumen (red arrow in A), which is completely unseen on EID-CTA. A 78-year-old male underwent PCD-CT coronary angiography (C), showing a moderate stenosis within a circumflex artery stent (red arrow in C). Abbreviations: CTA, CT angiography; EID, energy-integrating detector; PCD, photon-counting detector.

Blooming artifacts from partially calcified plaques and stents degrade image quality and diagnostic accuracy in conventional coronary CTA. Calcium blooming refers to the exaggerated appearance of calcified structures due to the scanner’s limited spatial resolution. The “blurring function” of the scanner operates on the bright signal from calcium and blurs the calcium beyond its actual boundaries. Similar blooming artifacts also occur with stents, where high signal from metallic struts obscures the true lumen, making the lumen appear falsely narrowed and limiting assessment of in-stent stenosis. Higher VMI energies, sharper kernels, larger matrix sizes, and smaller detector pixels in the ultra-high resolution (UHR) scan mode of PCD-CT significantly reduce calcium blooming (Figure 1).^17^^,^^18^ PCD-CT mitigates stent blooming artifacts by using thin (0.2 mm) slices, very sharp kernels, and a high matrix size (1024 × 1024), which are not achievable with conventional CTA (Figures 2–3).^19^

PCD-CTA decreases calcium blooming and improves assessment of luminal stenosis, as identified by invasive coronary angiography (ICA), including in challenging populations with a high prevalence of calcified plaques.^11^^,^^20–22^ CAD-RADS 2.0 is used to risk-stratify patients for future coronary events, relying primarily on luminal stenosis but also on the identification of imaging features of high-risk plaque, such as low-attenuation plaque and spotty calcifications (Figure 1). Spotty calcifications are defined as microcalcifications or punctate calcifications within fibroatheromas, are often seen with low attenuation plaque, and indicate a relatively higher risk of plaque rupture compared to other atherosclerotic calcifications.

The improved assessment of coronary PCD-CTA often results in reclassification of stenoses and CAD-RADS scores to a lower grade compared to conventional CT (up to 54% of patients;^9^^,^^17^ Figure 1), as well as detecting new luminal stenoses not identified at conventional CT (not infrequently, the most severe stenosis).^23^ High-resolution coronary PCD-CTA also improves luminal assessment within coronary stents (eg, in-stent stenosis) compared to conventional CT when using ICA as a reference.^12^^,^^24^^,^^25^

Although long-term outcomes regarding the clinical impact of coronary PCD-CTA are lacking, emerging evidence suggests meaningful downstream effects on patient management. One study involving more than 7800 patients who underwent ICA following PCD or conventional coronary CTA demonstrated that coronary PCD-CTA had significantly fewer referrals to ICA and higher intervention rates, in addition to improved accuracy.^10^

Beyond luminal assessment, coronary PCD-CTA enables improved evaluation of plaque burden and composition. A virtual non-iodine algorithm (PURECalcium; Siemens Healthcare) generates calcium scores from coronary PCD-CTA that correlate better with calcium scores from true noncontrast CT than do traditional virtual noncontrast images, but remains imperfect.^26^^,^^27^ Coronary PCD-CTA with 55-keV images can detect small, low-density plaques, even in patients with a coronary artery calcium score of zero.^28^ The improved spatial resolution of PCD-CT decreases total plaque volume and increases noncalcified plaque volume, with variable changes in calcified plaque volume.^29^^,^^30^ Moreover, PCD-CT VMI standardizes the CT number (in Hounsfield units) based on plaque composition analysis and improves reproducibility.^31^

### Plaque characterization

The improved spatial resolution of PCD-CT enables more detailed visualization of complex plaque with sharper boundary definition and greater sensitivity to smaller plaque components.^32^ Reduced partial volume averaging decreases calcium blooming and improves identification of noncalcified plaque (Figure 1).^29^^,^^33^ Several studies have shown good reproducibility and histopathologic correlation of PCD-CTA with features of plaque vulnerability, such as fibrous cap area and thickness, lipid-rich necrotic core, increased vasa vasorum density in carotid plaques, and greater ability of PCD-CTA to identify plaques with lipid-rich components and spotty calcifications.^33^

Plaque analysis with CT has traditionally relied on fixed CT-number thresholds to segment calcified, noncalcified, and low-attenuation noncalcified plaque volumes. For example, Vattay et al. applied thresholds (calcified plaque: >350 HU; noncalcified plaque: 30–350 HU; low-attenuation noncalcified plaque: −100 to 30 HU) in 51 human subjects.^34^ They found comparable noncalcified plaque volumes between the low-energy threshold images and 100–180 keV VMI reconstructions, but calcified plaque volume decreased steadily above 70 keV, whereas low-attenuation noncalcified plaque volume increased with increasing keV level. Thus, the use of VMIs without consensus guidelines to determine which energy levels and CT number thresholds to use is extremely problematic for accurate, reproducible measurements.

An alternative approach is a 3-material decomposition of CTA images to isolate calcium, fat, and fibrotic tissue. However, because fat and fibrotic tissue have similar effective atomic numbers, the amplified image noise inherent to 3-material decomposition further complicates reliable segmentation and quantification.

Artificial intelligence (AI)-based methods can also be used to detect high-risk plaques and reproducibly quantify coronary plaque burden, a predictor of major adverse cardiovascular events, within a clinically relevant time frame.^32^ Although expert consensus, medical society, and clinical practice recommendations remain lacking for standardized selection of VMI energy (keV) levels, in coronary CTA stenosis and plaque assessment, clinical experts generally recommend 90–100 keV VMIs to reduce calcium and stent artifacts and lower energy VMIs (eg, 40–65 keV) for noncalcified plaque and stenosis assessment.^35^

### Peripheral runoff and abdominopelvic CTA

**Figure 4:**
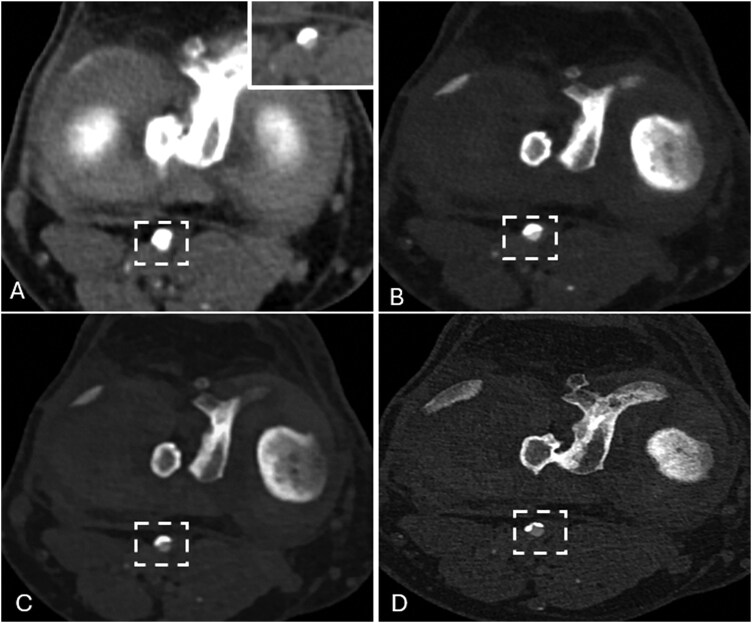
A 77-year-old male with calcified plaques in the popliteal artery underwent EID-CTA (A), demonstrating a calcific plaque with blooming artifact (white dashed box) that prevents accurate visualization of the lumen, even in a delayed phase (A inset). Same-day PCD-CT with different reconstructions shows calcium blooming artifact (white dashed box in B) with a smooth kernel (Qr48), with decreased blooming using a high-energy VMI (100 keV; white dashed box in C) even when using a smooth kernel; this is further reduced when a high-energy VMI (100 keV) is combined with a sharp, higher-resolution reconstruction kernel (Bv68; white dashed box in D). Abbreviations: Abbreviations: CTA, CT angiography; EID, energy-integrating detector; PCD, photon-counting detector; VMI, virtual mono-energetic images).

Runoff CTA provides an anatomic assessment for peripheral arterial disease diagnosis and revascularization planning,^36^^,^^37^ but is hampered by calcium blooming in the small leg vessels. Sharper reconstruction kernels and thinner slices at PCD-CTA significantly improve the evaluation of heavily calcified small-diameter vessels and decrease blooming artifact from calcified plaque and stents (Figure 3).^16^ Low-energy VMIs are used to increase iodine luminal signal, with simultaneously reconstructed high-energy VMIs used to further reduce calcium bloom (Figure 4).

**Figure 5:**
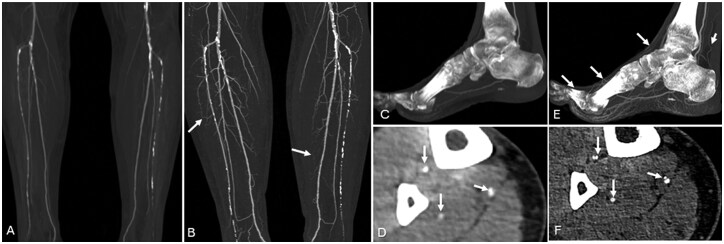
Runoff CT angiography of the lower extremities in 3 different patients. (A) EID-CT 3-dimensional (3D) maximum intensity projection (MIP) showing major lower-limb vessels. (B) PCD-CT 3D MIP from the same patient as (A), demonstrating improved visualization of perforating arteries and small branches. (C, E) 3D MIPs in another patient (C from EID-CT; E from PCD-CT) showing additional fine vessels on the dorsum of the foot not appreciated on the corresponding EID-CT. (D, F) Axial images (D from EID-CT; F from PCD-CT) highlighting small calcified arteries (arrows) with minimal distinction between calcification and lumen on EID-CT but clearly depicted on PCD-CT, with distinct separation of the contrast-filled lumen from calcifications, allowing accurate luminal assessment. Abbreviations: CTA, CT angiography; EID, energy-integrating detector; PCD, photon-counting detector.

Peripheral runoff PCD-CTA displays a larger number of small fibular perforators and fewer occlusions because of calcific plaque (as a small lumen can be visualized) compared to conventional energy-integrating detector (EID) CTA,^38^ despite the use of substantially less iodine contrast (Figure 5). The diagnostic accuracy of PCD-CTA in detecting stenosis >60% as identified by digital subtraction angiography is high, with sensitivity, specificity, and accuracy reported to be good to excellent (Table 2).^15^^,^^16^

**Figure 6:**
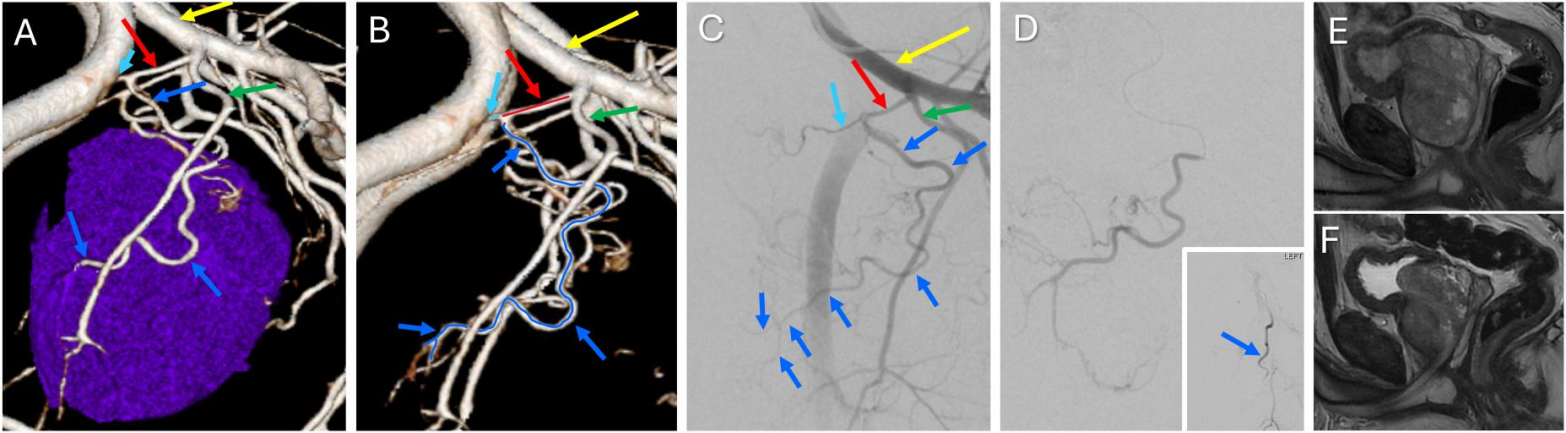
A 59-year-old male underwent preprocedural PCD-CTA for prostatic artery embolization. (A, B) High-resolution PCD-CTA 3D reconstructions (left pelvic oblique views) demonstrating the pelvic vasculature with the prostate gland segmented in purple (A). The left internal iliac artery (yellow arrows), the anterior division of the internal iliac artery (green arrows), and the vesicoprostatic trunk (red arrows; overlay in B) arise from the anterior division. Distal branching shows the left prostatic artery (blue arrows; overlay in B) and the superior vesical artery (light blue arrows; overlay in B). (C, D) Corresponding digital subtraction angiography (DSA) images obtained during the embolization procedure confirm the same arterial anatomy; the inset in D shows successful catheterization and embolization of the prostatic artery. (E, F) Sagittal T2-weighted MRI images before and after embolization, respectively, showing interval reduction in prostate volume from 155 mL (E) to 90 mL (F) following successful PAE. High spatial resolution of PCD-CTA enabled confident delineation of complex pelvic arterial anatomy that was overlaid on the DSA images during the procedure using embolization guidance software. Abbreviations: Abbreviations: CTA, CT angiography; DSA, digital subtraction angiography; EID, energy-integrating detector; PAE, prostatic artery embolization.

Within the abdomen, PCD-CTA has been shown to improve image quality (Figure S2) and radiologist confidence and agreement in assessment of vascular invasion in pancreatic cancer.^39^ PCD-CTA also better demonstrates the small arteries supplying the prostate and is used to plan and guide prostate embolization (Figure 6).^40^

### CSF-venous fistulas and venous imaging

The potential medical benefit of PCD-CT imaging of small veins is in its infancy, except for its role in diagnosing CVFs, which are abnormal, often transient connections between the spinal subarachnoid space and adjacent small intra- or paraspinal veins that result in unregulated CSF drainage into the venous system, leading to spontaneous intracranial hypotension. Identification of CVFs is clinically significant given their substantial morbidity and suboptimal detection at conventional (CT or dynamic) myelography, with precise localization guiding selection of targeted treatment options, including transvenous Onyx (Medtronic) embolization, surgery, or percutaneous fibrin glue injections for these abnormal venous connections.^41^

**Figure 7:**
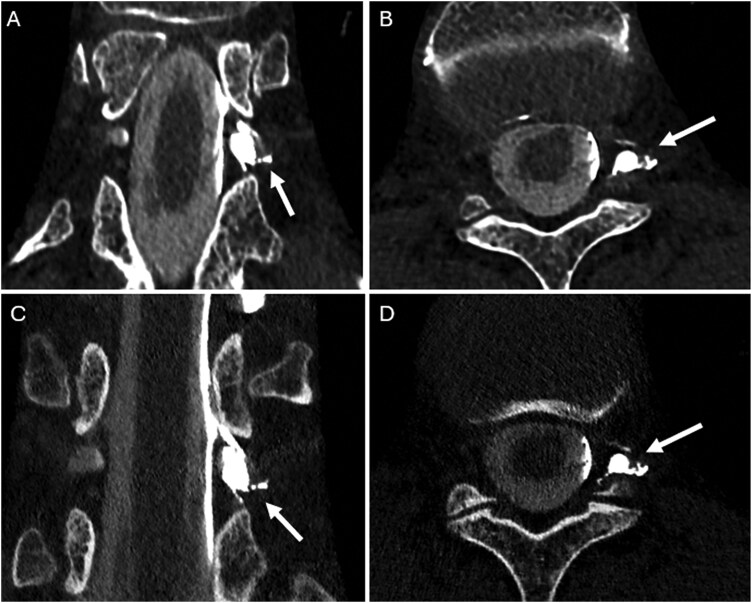
A 57-year-old female with a left T11–T12 CSF-venous fistula (white arrows in A–D), shown on coronal and axial images. The top row (A and B) shows high-spatial-resolution reconstructions using a sharp kernel (Qr89) combined with CNN denoising. The bottom row (C and D) shows a similar sharp kernel (Qr89) combined with the maximum strength of commercially available quantitative iterative reconstruction (QIR4). The CSF-venous fistula is apparent on all images, but the noise texture is markedly improved in the CNN-denoised Qr89. Abbreviations: CNN, convolutional neural network; QIR, quantitative iterative reconstruction.

PCD-CT myelography (PCD-CTM) uses thinner slices, UHR mode, and sharper kernels, and has demonstrated markedly higher sensitivity for detecting CVFs compared to conventional CT myelography (Figure 7; Table 2). Among patients with a low pretest probability of CVF based on a low Bern score (of 0–2; an MR-based prognostic score), PCD-CTM still detected definitive CVFs in 56% of patients (substantially higher than the anticipated 0–20% with conventional imaging modalities), with these patients undergoing subsequent embolization treatment, with resolution of intracranial hypotension symptoms.^42^

It is anticipated that PCD-CTM will continue to evolve by using ultrasharp reconstruction kernels (eg, Qr89) with AI-based denoising,^43^ with an increasing number of clinical studies showing its utility in this patient population.

**Figure 8:**
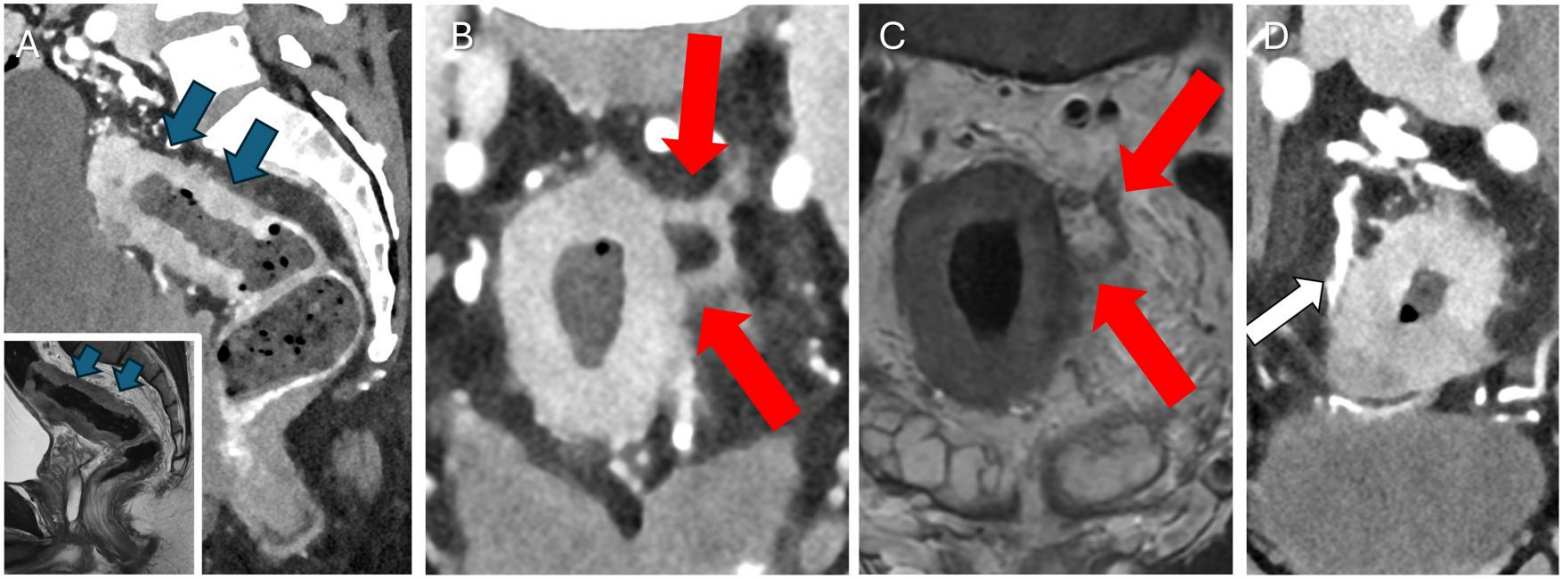
A 58-year-old male with mid-rectal cancer demonstrated on sagittal image from bolus-tracked, portal phase PCD-CT (A, blue arrows; corresponding MRI, inset). Coronal 40-keV PCD-CT image shows the circumferential cancer with a thick, enhancing wall and tumor extending into small left superior pararectal veins representing extramural venous invasion (B, red arrows), also shown on rectal cancer MRI later the same day (C, red arrows). Note that on portal phase 40-keV PCD-CT images arteries and veins are brightly opacified and appear white (D, white arrow) on another coronal image. Extramural venous invasion is an imaging feature of advanced rectal cancer and is associated with distant metastases, higher rates of local recurrence, and decreased survival. Abbreviation: PCD, photon-counting detector.

Imaging small veins is emerging in other body regions as well. PCD-CT can display rectal cancers with 1-mm 40-keV images and high levels of iterative reconstruction,^44^ and extramural venous invasion is not infrequently seen on PCD-CT (Figure 8). Extramural venous invasion is associated with distant metastases and local recurrence, so its recognition on CT can be useful to clinicians in guiding therapy and imaging surveillance.

### CTA of the head, neck, and intracranial arteries

PCD-CTA has shown substantial advantages over conventional EID-CT in imaging of small intracranial arteries. A prospective study comparing standard-resolution PCD-CTA (as a surrogate for conventional CT) to UHR PCD-CTA demonstrated that UHR PCD-CTA significantly enhances the detection and morphological assessment of unruptured intracranial aneurysms compared to standard-resolution reconstructions, improving display of aneurysm features and small adjacent branch vessels (Table 2).^13^ Another study showed that both standard-resolution and UHR (0.2-mm slices) PCD-CTA of intracranial vessels are preferred over conventional CTA, with a significant increase in diagnostic confidence.^45^

In craniocervical artery dissections, PCD-CTA improves the detection of pseudoaneurysms, subtle mural hematomas, intimal flaps, and intramural contrast compared to conventional CTA, particularly in the vertebral and internal carotid arteries, while decreasing radiation dose.^46^

For intracranial stents and flow diverters, PCD-CTA improves detection of in-stent stenosis while minimizing blooming and beam hardening artifacts by using sharp reconstruction kernels (Hv64 and Hv72) and thin 0.2-mm slices, an approach that increases diagnostic confidence.^47^

**Figure 9:**
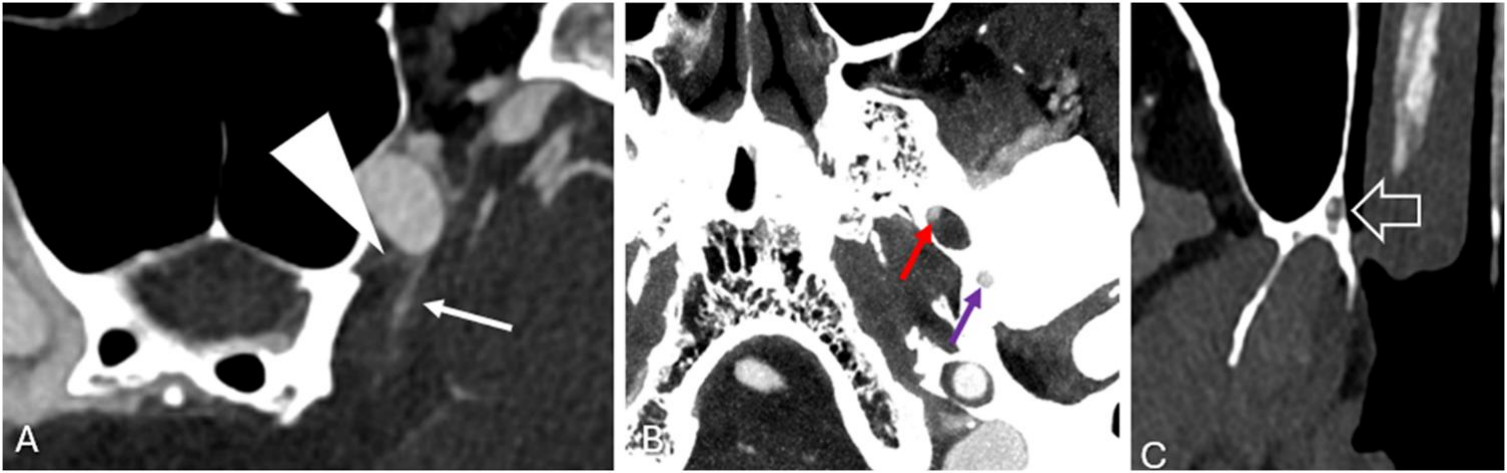
PCD-CTA axial images in (A) show meningohypophyseal trunk branches, including the inferior hypophyseal artery (white arrowhead) and the marginal tentorial artery (Bernasconi–Cassinari artery) supplying the tentorial meninges (white arrow). PCD-CTA in (B and C) shows small branches of the maxillary artery, including the artery of foramen rotundum (red arrow in B), the middle meningeal artery within the foramen spinosum (purple arrow in B), and the greater and lesser palatine arteries (white block arrow in C). Abbreviations: CTA, CT angiography; PCD, photon-counting detector.

Although EID-CTA permits excellent depiction of the larger intracranial vessels such as the vertebrobasilar system and circle-of-Willis and their proximal branches, PCD-CTA improves visualization of smaller intracranial arteries such as the meningohypophyseal trunk and maxillary artery and their branches (Figure 9), orbital arteries including the central retinal artery, as well as rare vascular anomalies (eg, aberrant internal carotid arteries).^48–50^ Technical advances are still needed to permit routine visualization of structures inconsistently seen with PCD-CT, eg, the blood supply to auditory structures, and the distal branches of the anterior inferior cerebellar artery (the labyrinthine and subarcuate arteries), which are generally not visible on EID-CTA.

### Pulmonary CTA and reduced iodine dose

**Figure 10:**
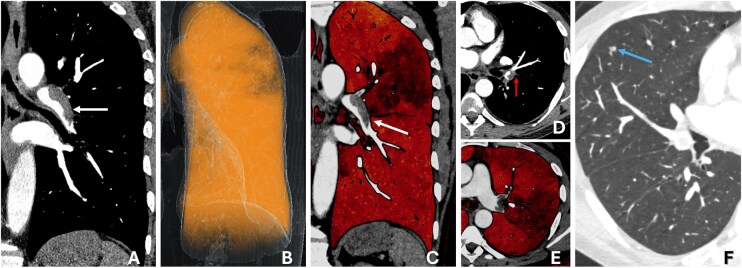
A 46-year-old male underwent a high-pitch multienergy pulmonary embolism (PE) study with PCD-CT showing acute bilateral pulmonary emboli, including a left lobar embolus (white arrows in A and C) causing complete occlusion of the superior lingular artery (red arrow in D). The overall embolic burden is demonstrated on the PCD-CT perfusion maps (reduced yellow color in B and focal dark-red perfusion defects in C and E), with multiple areas of hypoperfusion corresponding to vascular territories distal to the embolism. High-pitch acquisition reduces motion artifacts, permitting imaging of the chest in a single second, with a 0.6-mm slice thickness demonstrating a small 3-mm solid pulmonary nodule in the right middle lobe (blue arrow in F). Abbreviations: PCD, photon-counting detector; PE, pulmonary embolism.

Dual-source PCD-CTA permits high-pitch scanning (pitch 3.2) that reduces scan time (to about 1 second for the thorax) and reduces motion artifacts, which can be important for visualizing small vascular structures near the heart. Dual-source high-pitch PCD-CTA of the pulmonary arteries reduces radiation dose, improves iodine signal and image quality, and reduces motion artifacts compared to high-pitch low-tube potential conventional CTA or conventional dual-energy CTA.^51–54^ Dual-source high-pitch PCD-CTA may be particularly useful in an emergency setting and permits acquisition of high-quality images during free breathing (Figure 10).^52^ Kerber et al. have recently shown that iodine maps from PCD-CTA of the pulmonary arteries can be used to diagnose chronic thromboembolic disease with high accuracy, similar to dual-energy CT.^55^

The increased iodine contrast of PCD-CT and its ability to generate lower-energy VMIs (eg, 50 keV) increase iodine signal and contrast-to-noise ratio, allowing diagnostic imaging with reduced iodine dose compared to EID-CT. Studies have shown that PCD-CT can achieve diagnostic quality with substantially reduced contrast volume or salvage scans with suboptimal enhancement, particularly when low-energy VMIs are combined with high-pitch acquisitions to minimize motion artifacts.^56–59^

## Current limitations and challenges

*Technical limitations.* In the commercial PCD-CT system (Alpha class scanners, Siemens Healthineers) with the longest period of clinical use, several limitations have been identified. As other scanner manufacturers enter the market, different challenges or limitations may be identified in those systems. On Siemens systems, the higher spatial resolution mode uses a narrower collimation, requiring tradeoffs for some patients and diagnostic tasks, such as slower table speed. Another constraint is that tube power limits can make it difficult to use the required small focal spot size in large patients. Although PCD-CT has less image noise than conventional CT at the same spatial resolution and dose, the sharpest reconstruction kernels available on the scanner are not routinely used due to excessive image noise. For example, vascular protocols on the Siemens PCD-CT scanners typically use kernels such as Bv56 to Bv72 or Hv56 to Hv72, which provide lower spatial resolution than the scanner’s maximum capability but with a more favorable resolution-noise tradeoff.

Limitations also exist in spectral imaging. For example, with x-ray tube potentials below 120 kV, only a limited set of multienergy image types will be available because of insufficient spectral separation at lower tube potentials. A fundamental limitation of PCD technology, which uses high-atomic-number detectors (CdTe or CsZnTe), is spectral overlap across energy thresholds (bins) caused by non-ideal physical effects, such as charge sharing and k-escape. These effects reduce the spectral separation between the energy thresholds and limit multienergy capabilities. The use of low-atomic-number detectors (eg, Si) may mitigate this effect somewhat, but at the expense of spatial resolution.

Siemens has developed a multienergy algorithm that aims to remove all calcific-plaque contributions from a CT image while leaving all other pixels and CT values unchanged (PureLumen).^60^ However, processed images need to be viewed alongside high-resolution PCD-CT images to ensure fidelity to luminal assessment, with one coronary PCD-CTA study showing a 12% rate of erroneous plaque subtraction.^61^ This or similar algorithms may help determine stenoses in the presence of complex calcific plaques, thereby improving CT-derived fractional flow reserve estimates.^62^ Motion artifact remains a challenge even with dual-source coronary PCD-CTA in patients with high or irregular heart rates, along with segmentation errors and partial-volume averaging of complex plaque components.

*Operational challenges*. Despite its many advantages, deploying PCD-CT into clinical practice presents substantial operational challenges, principally the creation of new imaging protocols, but also integrating a new CT system and technology into an existing fleet, and managing downstream issues in image reconstruction and interpretation.

Using a PCD-CT system as if it were a conventional CT system negates its value. Protocols must be designed specifically for PCD-CT rather than copied from EID-CT, or its benefits will not be realized. PCD-CT systems produce lower image noise at the same spatial resolution and dose as conventional CT, despite detector pixel sizes that are typically 2–3 times smaller. This benefit is particularly evident with sharper kernels, where high resolution (or thinner slices) is achieved with less increase in noise.^63^^,^^64^ Therefore, sharper kernels are more advantageous for vascular imaging on PCD-CT, enabling improved image quality with reduced noise.

Optimal protocols often require sharper reconstruction kernels, larger matrices, and tailored scan modes for demanding vascular tasks. Table 3 summarizes how PCD-CT protocols are adapted for common vascular imaging tasks to overcome anticipated challenges. Appendix S1 provides example protocols from our clinical practice that illustrate these approaches. Designing and validating such PCD-CT protocols requires thoughtful planning and quality assessment. Operational challenges also arise when protocols are overly complex, as they can slow reconstruction and temporarily render the scanner idle, reducing patient throughput. Protocol teams must balance exploiting PCD-CT’s strengths with maintaining practice efficiency.

**Table 3:** Adaptations in PCD-CT protocols that address the anticipated challenges associated with common diagnostic tasks in vascular imaging at conventional (EID) CTA.

Diagnostic task or PCD-CT protocol	Anticipated challenge based on experience with conventional CT	Features of an optimized PCD-CT protocol in comparison to conventional CT for the same diagnostic task
Coronary CTA	Calcium blooming, beam-hardening from stents, motion, fast or irregular heart rate, small vessels	Multienergy UHR^a^ using 120- or 140-kV tube potential using dual-source acquisition Thin (0.2 mm) slices, high matrix (> 512 × 512) Sharp kernels are not available at conventional CT to minimize blooming artifacts and detect distal stenoses Consider low keV VMI to compensate for suboptimal opacification or low iodine dosage Consider high keV VMI to reduce blooming artifacts from calcium or stent Potential virtual non-iodine images to obtain coronary calcium score for risk stratification Automated algorithms to switch to different scan modes depending on heart rate and rhythm If triple rule out on a dual-source system, use multienergy dual-source high-pitch scanning but switch to 144 × 0.4-mm collimation for pitch > 3
Peripheral runoff CTA	Calcium blooming, small vessels, large volume coverage	Multienergy UHR using 120 or 140 kV tube potential Sharp kernels not available at conventional CT and thin (0.2 or 0.4 mm) slices with high matrix (> 512 × 512) to reduce blooming artifacts Consider using low-energy threshold (T3D) images rather than VMIs to speed reconstruction of a large number of images Consider delayed images to compensate for asymmetric perfusion
Detection of CSF-venous fistulas	Small and intermittent connection to a paraspinal vein, use of myelographic contrast	Multienergy UHR using 120- or 140-kV tube potential Sharp kernels not available at conventional CT with low energy VMIs to improve conspicuity of small intermittent CSF-venous fistulas High matrix (1024 × 1024)
Head and neck CTA	Calcium blooming, small and large vessels, adjacency to bone	Multienergy UHR using 120- or 140-kV tube potential 120 × 0.2-mm collimation Sharp kernels not available at conventional CT and thin (0.2 mm) slices to reduce blooming artifacts Low keV images to increase conspicuity of small vessels Special spectral image (SSI) file with a 0.4-mm slice thickness at 55 keV, which will permit creation of lower keV images (if needed)
Pulmonary CTA	Motion, potential need for decreased iodine, high cardiac output	Multienergy dual-source high-pitch scanning using 144 × 0.4-mm collimation for pitch 3.2 Use conventional kernels or slightly sharp kernels as focus is on iodine signal Reconstruct lower energy VMIs to increase iodine signal Consider routine reconstruction of iodine overlay and pulmonary blood volume images

Abbreviations: CTA, CT angiography; EID, energy-integrating detector; PCD, photon-counting detector; UHR, ultra-high resolution; VMI, virtual mono-energetic images.

a In PCD-CT, this implies a 120 × 0.2-mm detector configuration.

Most institutions operate a mixed CT fleet with multiple vendors and scanner generations, creating operational complexity for technologists. The Siemens PCD-CT system adds further challenges by introducing a new software platform and workflow. Because most practices have only 1 PCD-CT system, it occupies a small footprint within their scanner fleet. Practices must decide whether to staff PCD-CT with a small group of specially trained technologists or to train a broader pool that rotates to PCD-CT occasionally. Each approach has tradeoffs that may evolve as more scanners are added to the fleet.

Once images are reconstructed and sent to PACS, additional challenges emerge. PCD-CT studies can be more than 4 times the size of standard CT examinations, leading to lag during image scrolling and increased archival demands. Moreover, hanging protocols that were designed for conventional CT may not correctly recognize analogous PCD-CT image series, so PACS hanging protocols will likely need to be redesigned to facilitate efficient interpretation.

## New solutions and directions

**Figure 11:**
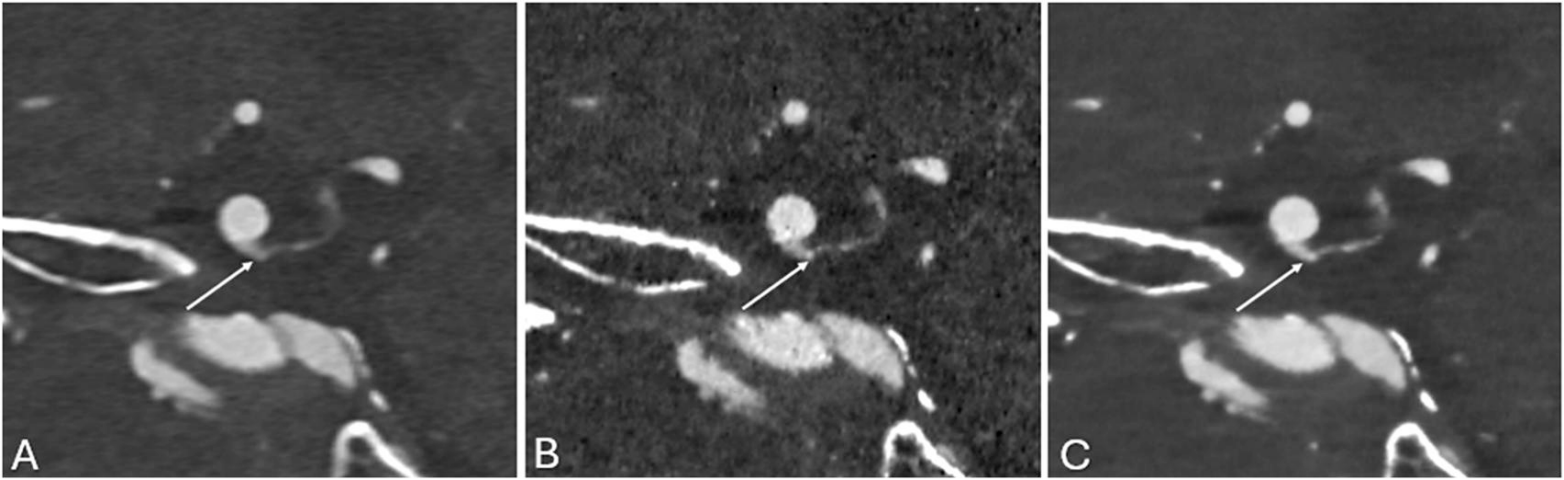
Three images are shown for a supraclinoid internal carotid artery (ICA) infundibulum (white arrows in A–C). The reconstructions used are: left (A), Hv56-QIR3; middle (B), Qr89-QIR4; and right (C), our novel CNN-denoised Qr89. The infundibulum is best demonstrated on the CNN-denoised Qr89 series. Abbreviations: ICA, internal carotid artery; CNN, convolutional neural network; QIR, quantitative iterative reconstruction.

*Technical.* Future technical improvements in PCD-CT are anticipated to address the current inability to use the sharpest, highest-resolution reconstruction kernels available at PCD-CT, as well as to reduce spectral overlap between energy bins. Increased image noise with sharp kernels is the primary limiting factor preventing routine vascular protocols from utilizing the highest-resolution kernels available at PCD-CT. To address this issue, noise reduction using nonlinear methods (eg, iterative reconstruction [IR] and deep-learning-based denoising and reconstruction [DLR]) has proven effective (Figure 11). IR methods may not be as effective as some DLR methods.^43^^,^^45^ Although DLR methods generally outperform IR for vascular imaging, clinical adoption requires careful validation to reduce potential issues such as instability and hallucinations. DLR methods have also been developed to reduce noise in multienergy material decomposition, where noise magnification is also a significant challenge.^65^

Future technical development to increase spectral separation in PCD-CT would substantially enhance multienergy capabilities. System optimization, including careful consideration of detector pixel size and balancing various effects such as pulse pileup and charge sharing, is an important aspect of PCD-CT. Innovative solutions to address charge-sharing effects, such as new Application Specific Integrated Circuit designs with charge-sharing correction mechanisms (eg, coincidence counting and charge summing) have been proposed in research settings.^66–69^

In addition to the system hardware, software algorithms to extract the most information from non-ideal PCD-CT data play a critical role. Material decomposition plays a critical role in multienergy CT, directly affecting image quality, quantitative accuracy, and precision. Advanced material decomposition algorithms have been actively investigated, including iterative-based and AI-based algorithms.^65^ Algorithms should be adaptive to the non-ideal spectral data acquired by PCD-CT to extract the maximum amount of information accurately and precisely. Additionally, algorithms should also have methods to control image noise arising from material decomposition to an acceptable level for clinical interpretation.

The Centers for Medicare & Medicaid Services recently approved commercial reimbursement for AI-based coronary plaque analysis using coronary CTA image data. This reimbursement will drive both continuing technical improvements in PCD-CT hardware and AI-based segmentation methods to identify and quantify imaging features of high-risk coronary plaques, as well as coronary plaque burden.

*Operational.* Many operational challenges will diminish as PCD-CT adoption increases, more clinical practices acquire multiple PCD-CT systems, and more CT system manufacturers offer commercial PCD-CT systems. Manufacturers will develop tools to assist in optimizing parameters for individual patients (eg, algorithms based on patient information: eg, heart rate, heart rate variability) to guide technologists in selecting acquisition modes/parameters. The radiology community will need to continue exploring new diagnostic tasks, particularly the benefits of imaging small arteries and veins, to capture the potential benefit of PCD-CT for patient care.

As awareness and access to PCD-CT grow, and patient and clinician demand outstrips capacity,^70^ requests for its use may exceed what institutions can accommodate. It is the responsibility of radiology practice leadership and expert clinical radiologists to make policy decisions regarding the exam types and the patients best served. Vascular indications are among the areas that, so far, have benefited most from PCD-CT, with justification to warrant prioritization for its use. Our CT practice uses a triage system to assign PCD-CT based on evidence and potential patient benefit, balancing requests from different subspecialties for PCD access by diagnostic task. Table 4 lists clinical indications and patient populations in vascular imaging that likely warrant triage to PCD-CT based on the evidence presented in this article.

**Table 4:** Clinical indications and patient populations in vascular imaging that warrant consideration of triage to PCD-CT based on current evidence.

Diagnostic task	Patient population(s)
Coronary CTA	Coronary stent High coronary artery calcium or known coronary calcifications Desire to minimize radiation dose and obtain concurrent coronary artery calcium score
Peripheral runoff CTA	Known or suspected peripheral artery disease
Detection of CSF-venous fistulas	Patients with prior negative conventional CT myelography or digital subtraction myelography Consider patients with low Bern score
Head and neck CTA	Known calcific plaques Exam performed for identification of small vessels or intracranial aneurysms
Pulmonary CTA	Hospitalized or emergency department patient Need for reduced iodine protocol

Abbreviations: CTA, CT angiography; PCD, photon-counting detector.

Subspecialty society and expert consensus recommendations for specific CT protocols for common diagnostic and quantitative tasks are anticipated and will facilitate adoption and performance assessment across institutions. For example, VMIs offer standardized CT numbers for a given VMI energy level, but VMI energy levels alter CT number values, which can affect plaque volumes when using CT number-based segmentation techniques.^34^ VMIs should ideally be used for plaque quantification owing to their inherent standardization capabilities, but medical societies will need to adopt new clinical standards and protocols.^31^ Clinical practice recommendations, which may arise from multicenter studies or expert consensus based on available evidence from single-institution studies, specifying appropriate VMI energy levels and optimal thresholds for deriving plaque volumes from photon-counting CT, are therefore mandatory and critical for multivendor, multisite radiology practices.

## Summary and conclusions

PCD-CT improves spatial resolution, reduces image noise at the same spatial resolution and dose, and has intrinsic multienergy capabilities, offering clear advantages over conventional CT for vascular imaging. These technical improvements translate directly into clinical benefits: visualization of small vessels across multiple body regions not seen with conventional CTA, improved display of critical vascular features that affect diagnosis and management, improved stenosis assessment in the presence of calcific plaque or stents by reducing blooming artifact, and improved characterization of complex plaque. Low-energy VMIs enhance iodine signal and contrast-to-noise ratio, enabling lower contrast doses and salvaging studies with suboptimal enhancement, whereas UHR modes allow detailed assessment of stents and fine vascular structures. Because PCD-CT has been available for only a relatively short time, we have emphasized diagnostic accuracy and efficacy studies as surrogates for long-term outcome studies, which are currently lacking.

For radiologists, the key to maximizing clinical impact lies in tailoring acquisition and reconstruction parameters to each diagnostic task. Tradeoffs, such as balancing higher spatial resolution and iodine signal with increased image noise and reduced volume coverage, must be considered in protocol design to display specific vascular territories and anatomic features. Early studies already demonstrate improved diagnostic confidence and accuracy across a range of diagnostic tasks in vascular imaging, with practical benefits for patient populations in which motion, calcific plaques, renal function, or small-vessel assessment are critical concerns.

In practice, PCD-CTA expands what is achievable with conventional CT angiography and is quickly becoming a new, valuable tool in daily vascular imaging. Broader adoption across institutions will require continuing technical innovation and development, consensus on imaging protocols, standardized thresholds for plaque and vessel assessment, and integration into multivendor workflows.

## Supplementary Material

umag021_Supplementary_Data

## Data Availability

There are no new data associated with this article.
