# Supplementary material for: Clinical benefits and current challenges of photon-counting detector CT in vascular imaging
Source: Radiol Adv. 2026 Apr 15;3(3):umag021. doi: 10.1093/radadv/umag021 (PMC13234556; doi:10.1093/radadv/umag021)
Supplement: umag021_Supplementary_Data [file umag021_supplementary_data.zip › Supplement_Data.zip!/supplementary file for review.v2.pdf]

Clinical benefits and current challenges of photon-counting detector CT in vascular imaging

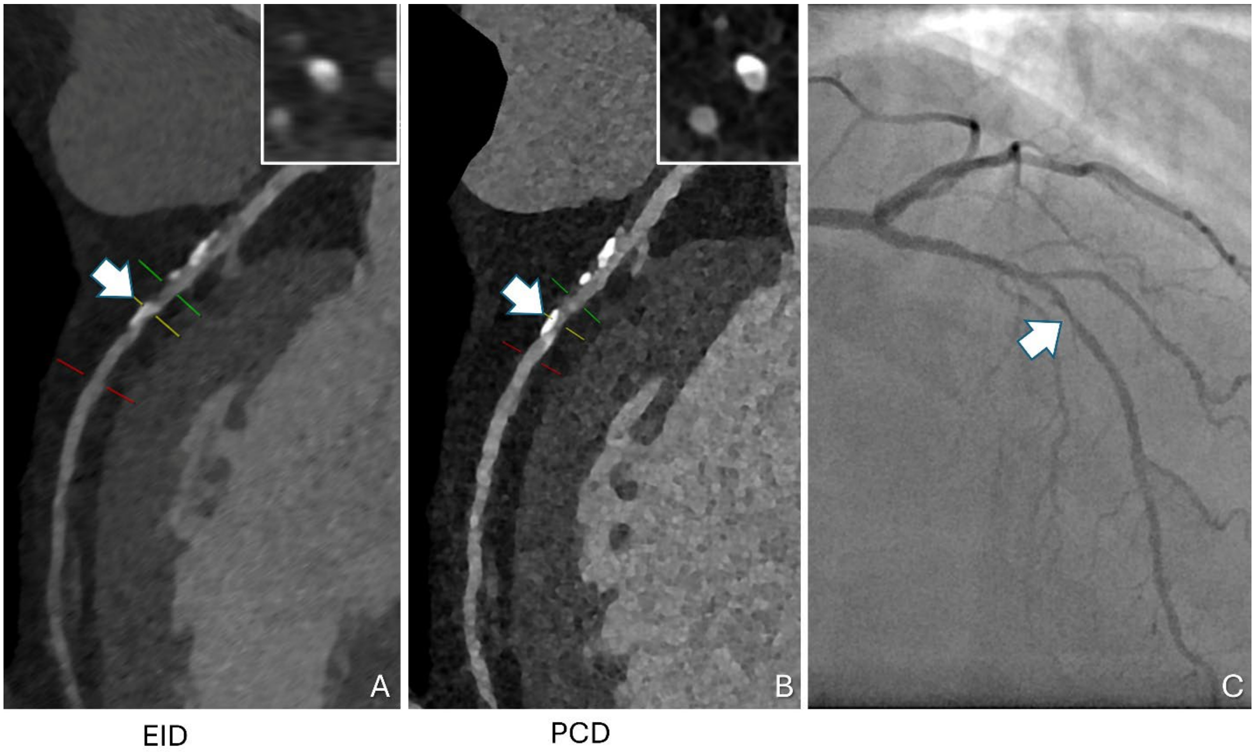

Figure S1. A 67-year-old male with curved planar reformats of the left anterior descending artery (LAD) obtained using EID-CT (A) and PCD-CT (B) on the same day, with coronary stenosis measured using a commercial quantitative coronary artery assessment tool (CT Coronary Analysis Workflow, syngo Via software version VB80A, Siemens Healthineers, Forchheim, Germany) using both modalities. At the mid-LAD segment (wide white arrows in A–C; and insets in A and B), stenosis severity differed between modalities: EID-CT estimated a 70–99% narrowing (CAD-RADS 4), whereas PCD-CT estimated a 50–69% narrowing (CAD-RADS 3). Invasive coronary angiography performed 24 days later (C) demonstrated a 60% stenosis in the same segment, aligning with the PCD-CT assessment.

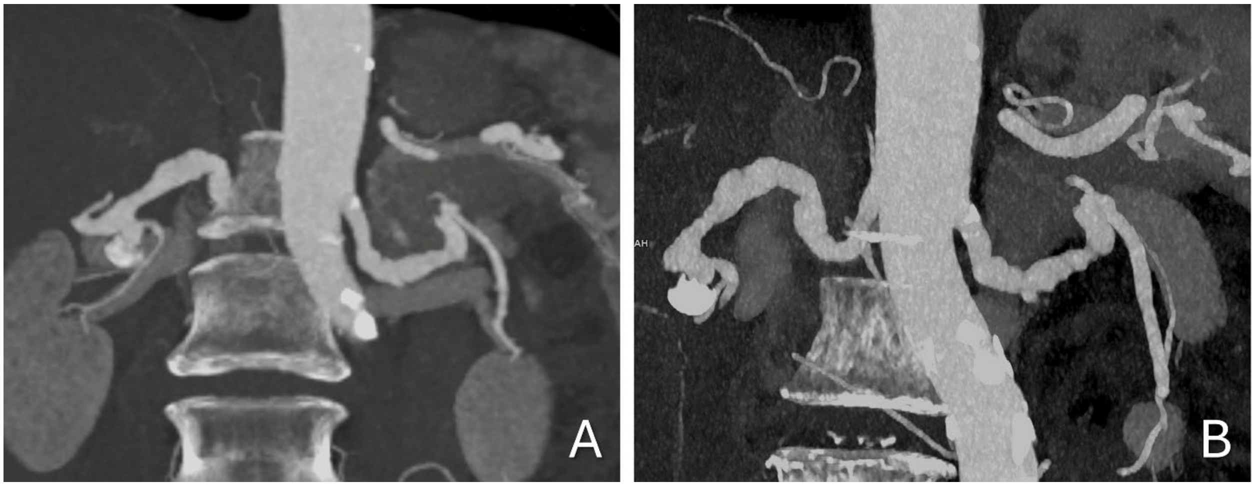

Figure S2. A 62-year-old female with fibromuscular dysplasia (FMD) involving the renal arteries. Photon-counting CT angiography (PCD-CTA) was performed with high-resolution parameters, including a sharp kernel (Bv56), 0.2-mm slice thickness, small field of view (250 mm), and high-matrix reconstruction (1024 × 1024) (B). A corresponding energy-integrating detector CT angiography (EID-CTA)–like reconstruction was also generated for comparison using a Bv40 kernel, maximum EID slice thickness of 0.6 mm, the same field of view (250 mm), and the maximum EID matrix (512 × 512) (A). PCD-CTA demonstrates enhanced visualization of the characteristic "string-of-beads" appearance and improved delineation of subtle luminal irregularities compared with the EID-like reconstruction. (FMD: fibromuscular dysplasia; PCD: photon-counting detector; EID: energy-integrating detector; CTA: CT angiography).

Supplemental Appendix One

Summary tables of example PCD-CTA protocols from our clinical practice. For comparison purposes we have included a column listing similar parameters from a dual-source 192-slice conventional CT system (Siemens FORCE; Siemens Healthineers, Malvern, PA) for the same protocols. In addition to items mentioned for these specific protocols, we generally reconstruct low-energy threshold (T3D) images as opposed to 70 keV images, as these are nearly identical in appearance and CT number given the mean x-ray tube energy of 67 keV at 120 kVp, but require less reconstruction time. Additionally, for all multi-energy PCD-CT protocols, a special spectral image series (SSI) is created (SPP on Siemens PCD-CT systems), which is an image format that provides information needed for spectral post-processing, so that another type of multi-energy image can be created (e.g., a VMI image with another energy level or an iodine map).

Table S1

Standard mode coronary CTA—Acquisition/Reconstruction Parameter	192-slice dual-source conventional CT	Dual-source PCD-CT
Scanner model	FORCE+	ALPHA*
Scan mode		CardiacDualsourceSpiralAdult AngioHighresultraQuantum¥
Pitch	Auto based HR	Auto based on HR
Rotation time	0.25 sec	0.25 sec
kVp	120 kV	120 kV
Quality reference mAs (QRM) or CARE keV IQ level	120	50@120 kV
Delay	Bolus tracking	Bolus tracking
Nominal (physical) collimation (mm)	192 (96) x 0.6	120 (120) x 0.2
Matrix	512x512	Automatically set; generally, 1024 x 1024
Reconstruction kernel	Bv40	Bv 60**
Iterative Reconstruction Strength	4	4
Slice thickness (mm)/reconstruction increment (mm)	0.6/0.3	0.2/0.1
Nominal $CTDI_{vol}$ (mGy)	21.0	27.3
**Summary of how PCD-CT protocol changes improve the diagnostic test**	Sharper kernel not available using conventional CT, thinner slices, and higher matrix reduce blooming artifacts from stents and calcific plaque, with the improved spatial resolution potentially detecting luminal stenoses not displayed by conventional coronary CTA. Note that clinical practice decided to perform high resolution coronary PCD-CTA with slightly higher radiation dose given improved resolution and image quality.	

* Neaotom ALPHA (Siemens Healthineers, Malvern, PA)
+ FORCE (Siemens Healthineers, Malvern, PA)
¥ Several options for coronary CTA are available, but this high resolution coronary CTA is the preferred imaging mode.
**For Siemens Healthcare, a higher number in the kernel name implies higher spatial resolution

Table S2

Pulmonary CTA for Pulmonary Embolism Detection Acquisition/Reconstruction Parameter	192-slice dual-source conventional CT	Dual-source PCD-CT*
Scanner model	FORCE[+]	ALPHA*
Scan mode	TurboFlash spiral[¥]	RoutineFlashSpiralAdultAngioBody Quantumplus
Pitch	3.0	3.2
kVp	120	120
Detector Configuration (physical collimation)	192x0.6 (96x0.6)	144x0.4 (144x0.4)
Rotation time	0.25 sec	0.25 sec
Quality reference mAs (QRM) or CARE keV IQ level	180	150
Prep Delay	Bolus tracking	Bolus tracking
Matrix	512 x 512	512 x 512
Reconstruction kernel/slice thickness (mm)/ increment (mm)	Br44 / 1.5/1.0<br>Qr54 / 0.75/0.5	Br44/ 1.5/1.0<br>Qr56/ 0.6/0.4
Iterative reconstruction	ADMIRE 3	QIR 3
Other image types<br><br>Image type kernel/slice (mm)/recon increment (mm)	None	Lung PBV<br>Qr40 / 1.5/1.0<br><br>SSI (Siemens SPP) series<br>QR40/1.0/0.8<br><br>VMI 40 keV (reconstructed if poor opacification of pulmonary arteries)<br>Qr40 / 1.5/1.0
Nominal $CTDI_{vol}$ (mGy)	7.6 mGy	6.6 mGy
**Summary of how PCD-CT protocol changes improve the diagnostic test**	$CTDI_{vol}$ of non-high-pitch pulmonary CTA exams in our clinical practice is 12 – 16 mGy; however, the high-pitch CT protocols are the preferred method if dual-source systems are available. The high pitch nearly eliminates all motion of small structures; use of the multi-energy mode (Quantumplus) permits reconstruction of 40 keV images when there is suboptimal opacification in the pulmonary arteries. The 60 keV images from the SSI image can be used retrospectively to obtain other multi-energy image types (e.g., VNC). The matrix size is kept constant at 512 x 512 to reduce image noise.	

* Neaotom ALPHA (Siemens Healthineers, Malvern, PA)
[+] FORCE (Siemens Healthineers, Malvern, PA)
[¥] "TurboFlash" is the manufacturer's designation for high-pitch technique.

Table S3

Lower extremity run-off CTA Reconstruction Acquisition/Parameter	192-slice dual-source conventional CT	PCD-CT
Scanner model	FORCE[+]	ALPHA*
Scan mode	Dual-energy[¥]	RoutineSpiralAdultAngioBody HighresultraQuantumplus
Pitch	0.4	0.5
kVp	Look up table for tube energy pairs based on patient size: 70/Sn150 for lateral size of 35 cm and below; 80/Sn150 for 36-40 cm; 90/Sn150 for 41-45 cm	120
Rotation time	0.25 sec	0.25 sec
Quality reference mAs/CAREkeV IQ level	Look up table for tube energy pairs<br>70/Sn150: 625/156<br>80/Sn150: 355/178<br>90/Sn150: 250/156	165
Prep Delay	Bolus tracking	Bolus tracking
Detector Configuration (physical collimation)	192x0.6 (96x0.6)	120x0.2 (120x0.2)
Matrix	512x512	Automatic (up to 1024x1024)
Reconstruction kernel/slice thickness/increment (mm)	Bv44 / 2/1.2<br>Bv44/ 1.0/0.5	Bv68 / 0.6/0.4<br>Bv44 / 1.0/0.5
Iterative Reconstruction	ADMIRE 2	QIR 3
Other image types	None	SSI (Siemens SPP) series at Qr40-3
Nominal $CTDI_{vol}$ (mGy)	12.84 mGy	12.0 mGy
**Summary of how PCD-CT protocol changes improve the diagnostic test**	Sharper kernel not available using conventional CT, thinner slices, and higher matrix reduce blooming artifacts from stents and calcific plaque, with the improved spatial resolution potentially detecting luminal stenoses not displayed by conventional CTA. Improved iodine signal from the detector combined with better spatial resolution displays small vessels not seen with conventional CTA.	

* Neaotom ALPHA (Siemens Healthineers, Malvern, PA)

[+] FORCE (Siemens Healthineers, Malvern, PA)

[¥] Preferred over single-energy protocol
